# Anatomical fit of the oneKNEE tibia design using statistical shape modeling

**DOI:** 10.1371/journal.pone.0354876

**Published:** 2026-07-31

**Authors:** Ingrid Dupraz, Yukihide Minoda, Nikolai Kornilov, Brigitte Altermann, Philipp Hauff, Arthur Bollinger, Berna Richter, Thomas M. Grupp

**Affiliations:** 1 Research and Development, Aesculap AG, Tuttlingen, Germany; 2 Department of Orthopaedic Surgery, Osaka Metropolitan University Graduate School of Medicine, Osaka, Japan; 3 Orthopedic Department N 17, Vreden National Medical Research Center of Traumatology and Orthopedics of the Ministry of Health of Russian Federation, Saint Petersburg, Russian Federation; 4 Department of Orthopaedic and Trauma Surgery, Musculoskeletal University Center Munich (MUM), Campus Grosshadern, LMU Munich, Munich, Germany; Southern Medical University Nanfang Hospital, CHINA

## Abstract

**Introduction and aim:**

Modern tibial implants should be designed to achieve an optimal anatomical fit. The aim of this study was to evaluate how well a newly developed tibial implant design (oneKNEE^®^ – B. Braun Aesculap, Tuttlingen, Germany) matches patient anatomy compared to three existing implant systems, using Statistical Shape Models (SSM).

**Method:**

SSMs for Caucasian and Asian populations were generated using CT scans of 120 Caucasian and 112 Asian osteoarthritic patients. Border anatomical variations were represented by individual anatomies from 14 Caucasian and 12 Asian patients. Tibial components were positioned in accordance with strict protocols established by senior knee surgeons (YM, NK). The anatomical fit was analyzed across all four implant systems in terms of antero-posterior and medio-lateral dimensions, tibial coverage and cortical bone support. Global tests for homogeneity and Dunnett’s tests were performed.

**Results:**

Bony coverage for Asian and Caucasian populations ranged from 82.9% to 88.4% for long-established designs and from 85.0% to 91.2% for more recently introduced designs. Among all designs, the oneKNEE^®^ implant demonstrated superior bony coverage and cortical support, followed by Attune^®^ (Depuy-Synthes, Warsaw, IN, USA), Columbus^®^ (B. Braun Aesculap), and PFC Sigma^®^ (Depuy-Synthes). For average anatomies, oneKNEE^®^ showed significantly better bony coverage and cortical score compared to Columbus^®^ and PFC Sigma^®^ (P < 0.01). In less average anatomies, oneKNEE^®^ exhibited statistically superior bony coverage compared to PFC Sigma^®^, although no significant difference was observed in cortical support.

**Conclusion:**

Compared with established tibial implant designs, the new design demonstrated improved virtual anatomical fit and coverage metrics based on computational modeling. These findings reflect a theoretical design‑level improvement and require biomechanical and clinical validation.

## Introduction

Optimal placement of the tibial component aims to achieve high tibial coverage while avoiding implant overhang and malrotation [1,2]. Proper tibial rotation is critical for knee kinematics and patellar tracking after total knee arthroplasty (TKA), with internal rotation being a risk factor for postoperative pain and poorer functional outcomes [2–6]. Rotational mismatch between femoral and tibial components greater than 10° further increases the risk of unfavorable outcomes [6].

The amount of cortical bone supporting the implant plays an important role in preventing subsidence of the tibial component [7]. Undersizing of the tibial implant on the medial and lateral sides can lead to subsidence and loosening of the tibial component [7–10]. Conversely, overhang of the tibial plateau—particularly on the medial and posterolateral sides—has been linked to clinical issues caused by soft tissue irritation [1,11]. An overhang in the medio-lateral (ML) dimension can lead to decreased flexion and pain [1,12–14]. Klasan concluded that upsizing within defined rotational safety zones resulted in the best patient-reported outcomes [11].

Tibial anatomy differs significantly among ethnic groups. In a study comparing Asian, African, and Caucasian bone models, significant differences were found in the antero-posterior (AP) dimension of the femur, trochlear depth, and AP/ML dimensions of the tibia [15]. A systematic review also highlighted ethnic differences in femoral and tibial dimensions, with white patients showing larger femoral AP dimensions than East Asians [16]. In another Statistical Shape Model (SSM) analysis, a global population was compared to bone models of Asian and Caucasian populations. The Caucasian SSMs were wider in ML, the male Asian SSMs were wider in AP, and the intercondylar notch of the female Asian SSMs was narrower [17]. Several studies report that current implant designs do not adequately account for these anatomical differences [18–21].

Symmetric and asymmetric tibial designs differ in coverage and practicality. While numerous studies have demonstrated higher bone coverage percentages for asymmetric tibial designs compared to symmetric designs [22–24], asymmetric designs tend to lead to posterolateral and posteromedial overhang [8]. Other studies conclude that symmetric tibial designs provide better coverage [25,26], suggesting that size range and actual shape are the most critical factors in tibial coverage [27].

The oneKNEE® tibial design was developed to achieve broad anatomical coverage across Caucasian and Asian patient populations while maintaining a limited number of size variants. This study focuses on the evaluation of exclusively virtual anatomical fit parameters, specifically tibial fit, coverage, and cortical bone support, in these populations, in comparison with three established implant systems, PFC Sigma^®^, Attune^®^ and Columbus^®^. PFC Sigma^®^ was the most widely used implant design in the NJR registry report 2023 (n = 398 426). Both PFC Sigma^®^ and the Columbus^®^ implant have been on the market for over 20 years. Attune^®^ is a design from the same manufacturer as PFC Sigma^®^ and represents a modern design comparable to the oneKNEE^®^ design evaluated in this paper.

Verifying the anatomical compatibility of implant designs using patient-specific CT data requires substantial effort and access to extensive bone databases. Statistical shape models represent a population’s anatomy and provide an efficient tool to evaluate the anatomical fit of TKA implants by encapsulating a large segment of the population into a limited number of three-dimensional models [17]. In this study, this approach is extended by combining population-specific statistical shape models with the targeted analysis of selected border anatomies for a systematic comparison of tibial implant designs.

The purpose of this study was to evaluate the anatomical fit of the oneKNEE^®^ tibial design and compare it to three other widely used designs. We analyzed Asian and Caucasian populations with respect to AP/ML dimensions, tibial coverage, and cortical bone support.

## Methods

### SSMs included in the analysis

The CT-scans from osteoarthritic patients with Kellgren–Lawrence grades one to three [28] were used for this analysis. The datasets were obtained from patients for whom a standard total knee arthroplasty was planned based on clinical assessment. Patients presenting with severe deformities or advanced arthritic anatomies requiring complex reconstruction techniques or customized implant solutions were not included, reflecting routine clinical practice. Accordingly, the analysed cohort represents patients suitable for standard TKA and does not include extreme deformities or advanced arthritic cases. A total of 120 Caucasian anatomies (65 female, 55 male, mean age: 68 ± 10 years; range 46–91 years) and 112 Asian anatomies (75 female, 37 male, mean age 65 ± 12 years, range 29–87 years) were included. Images were segmented and three-dimensional bone models were reconstructed for each patient (Materialise^®^, Leuven, Belgium). Written advice was obtained from the Ethics Committee of the Baden-Wuerttemberg State Medical Association, which confirmed that formal ethics approval was not required under Section 15(1) of the Professional Code of Conduct.

The following reference system was employed: The proximo-distal (PD) axis of the tibia was defined as the axis connecting the midpoint of the malleoli to the midpoint of the tibial spines. The AP axis was determined using the Akagi Line, connecting the medial third of the tuberosity to the center of the posterior cruciate ligament attachment [29], which was projected on the axial plane (orthogonal to the PD axis). The ML axis was the resulting orthogonal axis to the AP and PD axes.

The Caucasian SSM was constructed using 120 Caucasian anatomies, while the Asian SSM was based on 112 Asian anatomies. Seven bones in different sizes were derived from the Caucasian SSM and five sizes from the Asian SSM. Model validation was conducted as described by Dupraz et al. [17]. The selection of bone models reflects the patient population targeted for future treatment with this implant.

The tibia was virtually resected 6 mm below the deepest point of the healthy plateau (assuming a cartilage thickness of 2 mm), perpendicular to the mechanical axis. For two Asian and five Caucasian patient bone models, an additional 1–4 mm had to be removed to achieve a uniform resected surface. Remaining osteophytes were manually excised.

### Measurement of the AP/ML dimension

**Fig 1:**
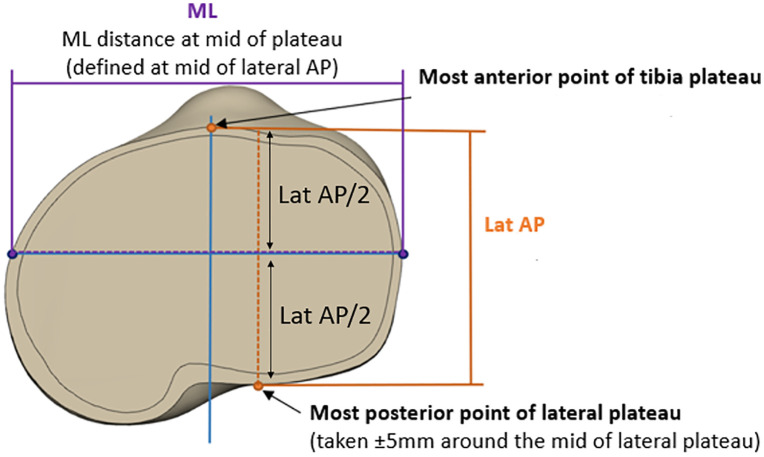
Definition of the AP and ML dimensions of the native tibia plateau.

**Fig 2:**
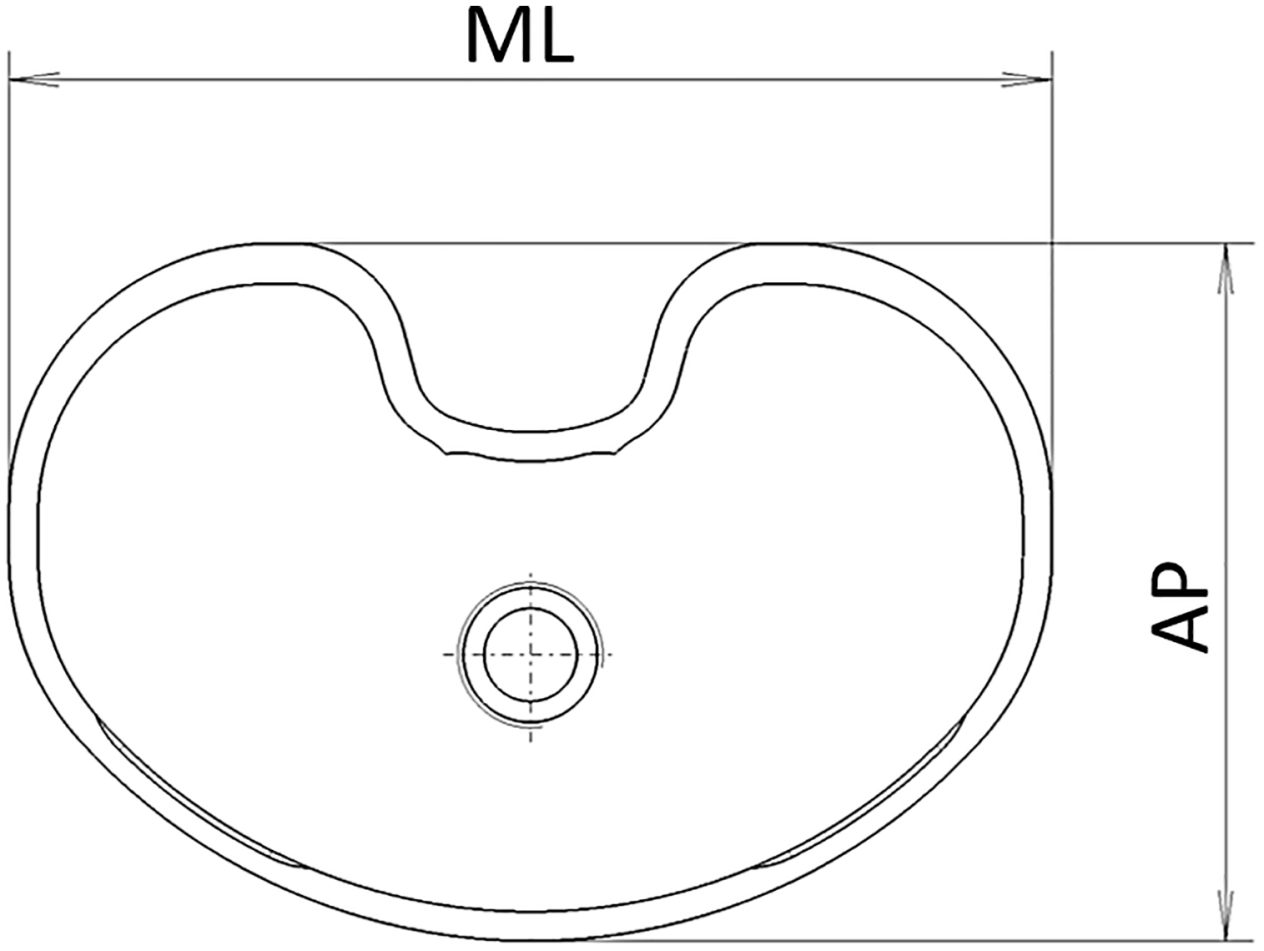
Definition of the AP and ML dimensions of tibia implants.

The AP dimension of the tibial plateau *Lat_AP* (Fig 1) was measured from the most anterior point of the total plateau to the most posterior point of the lateral plateau, within a range of ±5 mm around the midpoint of the lateral plateau. The lateral plateau was chosen as it is smaller than the medial plateau and therefore represents the critical dimension to consider for implant fit. The corresponding ML dimension was measured at the midpoint of *Lat_AP*. The AP/ML dimensions of the implants were defined as the maximum AP and ML dimensions (Fig 2).

### Patient bone models included in the analysis

Within this clinically relevant cohort, selected border anatomies were identified to represent extreme anteroposterior and mediolateral dimensions while remaining compatible with standard implant placement. Individual anatomies were selected from the 232 available anatomies according to the following method: the available AP dimensions (40 mm-60 mm) were stratified into eight consecutive AP size groups of 2.5 mm each. Within each AP size group, the Caucasian and the Asian patients with the smallest and largest ML dimensions were selected. In the event that, within an AP size group, all patients had a smaller or a larger ML dimension than the SSM, only one patient with the most extreme ML dimension was selected. Two Asian and two Caucasian patients with extreme proximal tibia anatomies were excluded, as their anatomies did not allow for a clinically acceptable placement of the implants according to the defined criteria. These patients were replaced by those with the closest AP/ML dimensions. In total, 14 Caucasian and 12 Asian individual anatomies were included in the analysis.

### Virtual implantation

Using three-dimensional bone models and implants, a virtual implantation was simulated clinically on the computer. The surgeon placed the tibial implant in a simulated surgical setting. The following implant designs were evaluated across all bone models: oneKNEE^®^ (B. Braun Aesculap, Tuttlingen, Germany); Attune^®^ (Depuy-Synthes, Warsaw, IN, USA); Columbus^®^ (B. Braun Aesculap); and PFC Sigma^®^ (Depuy-Synthes). The AP/ML dimensions of these implant designs are compiled in S1 Fig.

The tibia implants were positioned on each bone model. Tibial rotation was determined by the best-fit concept while remaining within a safe range compared to the Akagi line [29]. Starting from an initial rotation of 0°, the clinical authors introduced incremental tibial rotation to optimize bony coverage, according to the following rules: overhang was tolerated up to 1.5 mm laterally/medially and up to 0.5 mm anteriorly/postero-laterally. The components were internally rotated by up to 6° to stay within the safe zone [7,11,30]. The placement of each component on Asian and Caucasian bones was validated by respectively an Asian (YM) and Caucasian (NK) senior knee surgeon.

### Assessment of bony coverage and cortical support

The bony coverage was calculated as the percentage of bone surface covered by the implant. As the notch area is clinically irrelevant for bony coverage, the notch was artificially closed for all implants (S2 Fig).

A semi‑quantitative cortical bone support score was used to facilitate a structured and consistent comparison of tibial implant designs across different anatomies. The score was based on visual assessment of implant–bone contact in predefined regions and was applied uniformly across all implant systems to support relative comparisons of cortical support within the context of a virtual fit analysis. The tibial plateau was divided into four anatomical quadrants, and each quadrant was visually scored from 0–3 based on the presence and quality of cortical rim contact. The total cortical support score was defined as the sum of the quadrant scores (S3 Fig). This method is designed to closely approximate the way rim support is assessed intraoperatively by surgeons, thereby providing a realistic and reproducible basis for consistent comparative analyses across implant designs and anatomical morphologies.

### Statistical analysis

Average and standard deviation of bony coverage and cortical scores were computed for each implant and population. Mixed linear regression models compared the four implant systems, with implant and patient population (Caucasian/Asian) as factors.

The main analysis used the SSM as a model entity, i.e., the implant fit measures were calculated for individual SSMs. In the sensitivity analysis, individual patients with borderline anatomies represented the model entities. This approach yielded four models: bony coverage in the SSM population, bony coverage in borderline anatomies, cortical score in the SSM population, and cortical score in borderline anatomies.

As the interaction term system/population was not significant to the 0.05 level in any of these models, it was not included in the model report. In addition to the global tests for homogeneity of bony coverage and cortical score for the different implant systems and populations, Dunnett’s tests compared oneKNEE^®^ to the other designs; adjusted p-values are reported [31,32]. In addition to p-values, the magnitude of between-system differences was interpreted using absolute differences (percentage points) and 95% confidence intervals. Although the number of border anatomies included in the sensitivity analysis was limited, these cases were selected to represent extreme AP/ML dimensions within clinically relevant ranges and were intended to support comparative rather than population level conclusions.

## Results

### AP/ML Fit

**Fig 3:**
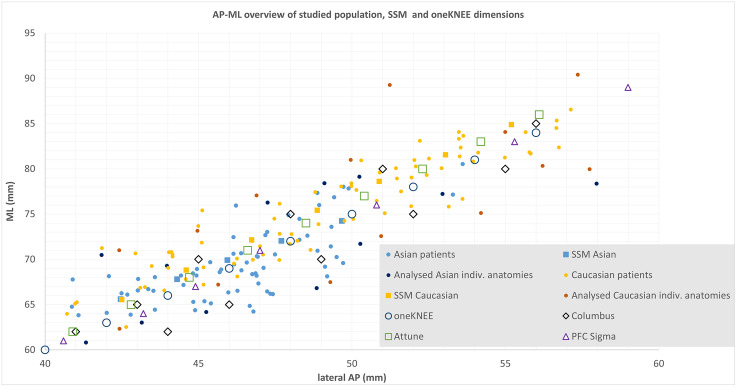
AP/ML dimensions measured on bone models (patients, SSMs) and AP/ML implant dimensions (oneKNEE®, Attune®, PFC Sigma®, Columbus®).

Fig 3 shows the AP/ML dimensions of the Asian and Caucasian patients, the five resulting Asian SSM sizes, the seven resulting Caucasian SSM sizes, and the 14 Caucasian and 12 Asian individual patient anatomies selected to represent less common populations. The dimensions of the oneKNEE^®^ implant sizes are evenly distributed in the middle of the cloud of points representing both populations (Fig 3).

### Bony coverage and cortical support

**Fig 4:**
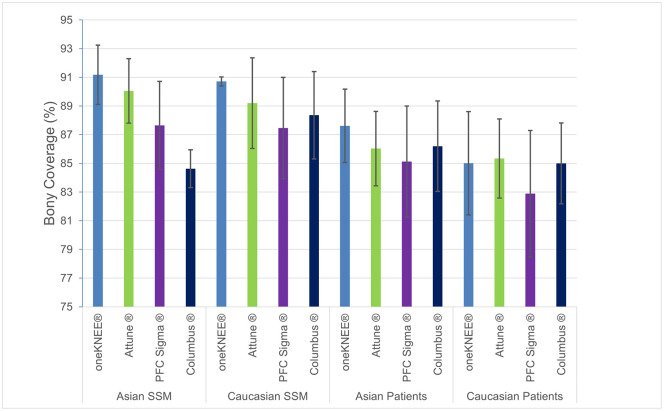
Bony coverage of all implant designs on the Asian SSM, Caucasian SSM, Asian Patients and Caucasian Patients (average, ±1 standard deviation). Displayed to visualize the distribution of values; quantitative comparisons are based on the full statistical analysis.

**Fig 5:**
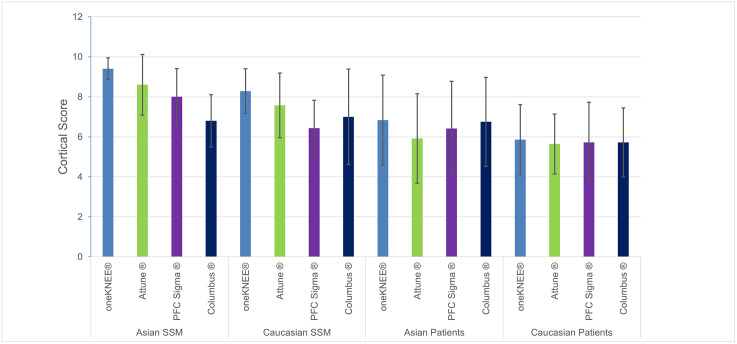
Cortical score of all implant designs on the Asian SSM, Caucasian SSM, Asian Patients and Caucasian Patients (average, ±1 standard deviation). Displayed to visualize the distribution of values; quantitative comparisons are based on the full statistical analysis.

The bony coverage and cortical scores for all systems and all populations are represented in Figs 4 and 5. Figures illustrating implant positioning and cortical support are provided as representative examples. The adjusted P-values of the Dunnett’s test are provided in Table 1. Absolute between-system differences (percentage points) and 95% confidence intervals are reported to facilitate interpretation (see Statistical Analysis and Table 2).

**Table 1:** Adjusted P-value for individual comparisons of oneKNEE^®^ to other designs (Dunnett’s test).

Adjusted P-Value (Dunnet’s Test)	Bony Coverage		Cortical Score	
	Average Anatomies (SSMs)	Border Anatomies (Patients)	Average Anatomies (SSMs)	Border Anatomies (Patients)
Attune^®^	0.3581	0.7071	0.325	0.0641
PFC Sigma^®^	0.0032	0.0013	0.0062	0.5258
Columbus^®^	0.0004	0.5852	0.0025	0.9282

**Table 2:** Bony coverage results of tibial implant designs based on Statistical Shape Models (SSM), reported as mean values with 95% confidence intervals.

Population	Implant	Mean %	95% CI low	95% CI high
Asian (SSM)	Columbus^®^	84.4	82.2	86.5
Asian (SSM)	Attune^®^	90.3	86.3	94.3
Asian (SSM)	PFC Sigma^®^	88.5	84.0	93.0
Asian (SSM)	oneKNEE^®^	91.3	87.6	95.1
				
Caucasian (SSM)	Columbus^®^	87.6	84.9	90.3
Caucasian (SSM)	Attune^®^	88.3	86.0	90.6
Caucasian (SSM)	PFC Sigma^®^	86.8	83.3	90.2
Caucasian (SSM)	oneKNEE^®^	90.7	90.4	91.1

The bony coverage in the Asian and Caucasian populations ranged from 82.9% to 88.4% for the PFC Sigma^®^ and Columbus^®^ implants and from 85.0% to 91.2% for the Attune^®^ and oneKNEE^®^ implants. For all designs, except Columbus^®^, the bony coverage was slightly better in the Asian population compared to the Caucasian population, although this difference was not statistically significant. Selected SSM comparisons (percentage-point differences) are provided in Table 2. For example, in the SSM analysis oneKNEE^®^ increased mean bony coverage by 6.9 percentage points versus PFC Sigma^®^ (91.3% vs 84.4%) and by 2.9 percentage points versus Columbus^®^ (91.3% vs 88.4%) in the Asian SSM; in the Caucasian SSM, the corresponding differences were 4.0 percentage points (90.7% vs 86.8%) and 3.1 percentage points (90.7% vs 87.6%).

The cortical support was highest for the oneKNEE^®^ design, followed by the Attune^®^ design. Notably, the oneKNEE^®^ design achieved an excellent fit of the anterior and posterolateral contours on the Asian SSMs, as shown in S4 Fig.

The model assessing bony coverage and cortical support demonstrated significant differences between the designs, as illustrated in Fig 4 and summarized in Table 1 and 2. However, the influence of population on bony coverage was not statistically significant.

Overall, the oneKNEE^®^ design exhibited the greatest bony coverage and cortical support, followed by the Attune^®^, Columbus^®^, and PFC Sigma^®^ designs (Table 2). For average anatomies (SSMs), oneKNEE^®^ showed significantly superior bony coverage and cortical scores compared to Columbus^®^ and PFC Sigma^®^ (P < 0.01). For border anatomies (individual patients), the bony coverage of oneKNEE^®^ was statistically higher than that of PFC Sigma^®^, whereas no significant differences in cortical support were observed between oneKNEE^®^ and the other designs.

## Discussion

This study evaluated the anatomical fit of two recently introduced and two clinically well-established tibial designs in Asian and Caucasian populations, using both average (SSM) and individual border anatomies. All designs demonstrated good bony coverage (>85%), with modern designs showing an improved fit to AP and ML dimensions in Asian populations compared to long-established designs.

Several studies have examined the AP/ML dimensions of the Asian population [18,19,33–38]. Our population aligns with published data but shows greater variability than Chinese [19] and Thai [18] populations. The Asian population of this study tended to be smaller in ML compared to the Chinese population studied by Li [33], while it appeared slightly wider in ML compared to the Chinese population examined by Cheng (94 males, 78 females) [19]. The Asian population of this study (75 female, 37 male) tends to be in the lower ML and upper AP range of the female Korean population and in the lower ML range of the Korean male population [35]. The studied Asian population corresponds generally with other published data but does not encompass larger Japanese male anatomies [37], which are better represented by the Caucasian dimensions in the present study. Compared to the Caucasian population studied by Li [33] (61 male, 87 female), the population in this study demonstrates broader variability, including more anatomies in the upper AP/ML range. Differences across studies reflect ethnicity and measurement definitions; here, AP/ML dimensions were defined to match tibial implant components.

Key factors influencing anatomical fit include implant shape, number and distribution of size increments, and component alignment. Given the wide variability in AP/ML dimensions within Asian and Caucasian populations, selecting the appropriate implant sizes and size increments remains a challenge. Modern designs (Attune^®^, oneKNEE^®^) showed the best match across populations. Although the number of available size increments has been identified as a key factor influencing anatomical fit [27], this study emphasized that an optimal distribution of sizes plays an even more critical role: the Columbus^®^ system did not achieve optimal coverage despite offering 11 sizes (6 main sizes plus 5 additional sizes with a larger AP dimension). In contrast, linearly distributed implant systems like Attune^®^ and oneKNEE^®^, which feature nine sizes, achieved better coverage.

Recently introduced designs better fit anatomy due to higher ML/AP ratios, enhancing cortical contact, and the anterior curvature of oneKNEE^®^ improved anterior support, particularly in Asian anatomies.

The alignment of the components was determined according to stringent criteria. Rotation of up to 6° was permitted, reflecting clinical practice and established evidence of positive clinical outcomes [11]. For nearly all cases 6° of internal rotation resulted in enhanced bony coverage. This improvement can be attributed to the symmetrical shape of the implant components combined with the asymmetry of the bone anatomy. However, it differs from previous findings from Clary [26], whose findings suggested minimal impact of tibial rotation on bony coverage. When rotating the Attune^®^ and Sigma^®^ implants to maximize bony coverage Clary obtained an average of 3–4° of internal rotation with high standard deviations between 4.4° and 5.1°. Additionally, Clary noted that tibiae exhibiting higher skew, smaller ML dimensions, and elongated medial plateaus necessitate greater implant plate rotation.

The results of our study, demonstrating bony coverage for recently introduced designs ranging from 85.0% to 91.2%, align with the findings of Dai [39], who reported an average bony coverage of 85–92%. In comparison, Clary and Wernecke [8,26] observed lower bony coverage, ranging from 80–84% and 80–88%, respectively. For the PFC Sigma^®^ design, Dai reported approximately 86% coverage for both Caucasian and Asian populations (estimated from a graph), whereas Clary reported 80.2% coverage primarily for Caucasian patients, and Wernecke reported 85%. Regarding the Attune^®^ design, Clary observed a coverage rate of 83.8%. These discrepancies can be explained by differences in component placement and methodologies for excluding the influence of the notch area. Dai allowed for ±5° of rotation around the neutral rotational alignment axis, while Clary and Wernecke perfectly aligned the component to the medial third of the tibia tubercle. However, even when optimizing component rotation for maximum coverage, Clary reported 81.7% coverage for PFC Sigma^®^ and 85.6% for Attune^®^. The integration of the notch area by Clary and Wernecke also contributed to lower coverage values compared to the results of this study. Similar to Dai, we excluded the notch area from the analysis. Dai excluded a pie-shaped region of the posterior notch, whereas in this study, the contour of each design was closed around the notch area to minimize its influence on bony coverage.

The clinical relevance of small differences in overall coverage remains unclear [26]. Accordingly, statistical superiority (e.g., p < 0.05) was interpreted as evidence of a difference between designs, whereas clinical meaningfulness cannot be inferred from significance alone and would require clinical validation. However, anterior coverage appears particularly important [26], which was successfully achieved with the anterior curvature of the oneKNEE^®^ design.

The limitations of this study are the following: The SSMs used in the study were based on 120 Caucasian and 112 Asian anatomies. While this sample size was adequate to ensure a reasonable representation of the populations, including a larger number of anatomies would enhance the representativeness of the models. Components were placed manually according to strict rules to allow objective comparison across designs. Interobserver and intraobserver reliability for implant positioning and cortical support scoring were not formally assessed. This approach might not fully reflect clinical practice, as surgeons may apply different rules to different anatomies which might be a limitation of the present study. Finally, quantifying the quality of cortical support is challenging and has not been proposed so far. In this study, cortical support was evaluated semi-quantitatively. Development of a fully automated algorithm was not pursued, as it was unlikely to yield clinically relevant differences. Overall, the findings are limited to virtual fit metrics and potential operator dependence in component placement, with no validation against biomechanical or clinical outcomes. The strength of this study lies in its use of both statistical shape models and individual border anatomies to evaluate the anatomical fit of implants for both average and border anatomies. While previous studies have examined anatomical fit across large patient cohorts, which necessitated fully automated component placement, this study employed manual placement by experienced knee surgeons for a limited number of anatomies.

In conclusion, this study introduces a novel methodology for evaluating the anatomical fit of implants within a given population by utilizing both statistical shape models and individual border anatomies. Compared with established tibial implant designs, the new design demonstrated improved virtual anatomical fit and coverage metrics based on computational modeling. These promising findings reflect a theoretical, design-level improvement and require further clinical validation.

## Supporting information

S1 Fig Tibia implant dimensions. (TIF)

S2 Fig Tibia implant with closed notch (used to compute bony coverage). (TIF)

S3 Fig Definition of scores to assess the quality of cortical support. (TIF)

S4 Fig Comparison of cortical rim support for the four tibial implant designs on the Asian XL SSM. The semi-quantitative score (0–3) is shown for each of the four anatomical quadrants. Representative example shown for illustration; conclusions are drawn from the summarized results across all models.
(TIF)
