# Supplementary figures and images for: Anatomical fit of the oneKNEE tibia design using statistical shape modeling
Source: PLoS One. 2026 Jul 31;21(7):e0354876. doi: 10.1371/journal.pone.0354876 (PMC13426962; doi:10.1371/journal.pone.0354876)

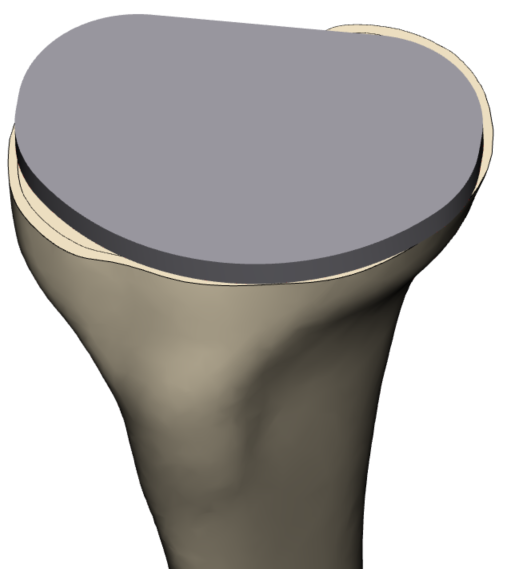

Supplement: S2 Fig — (TIF) [file pone.0354876.s002.tif]

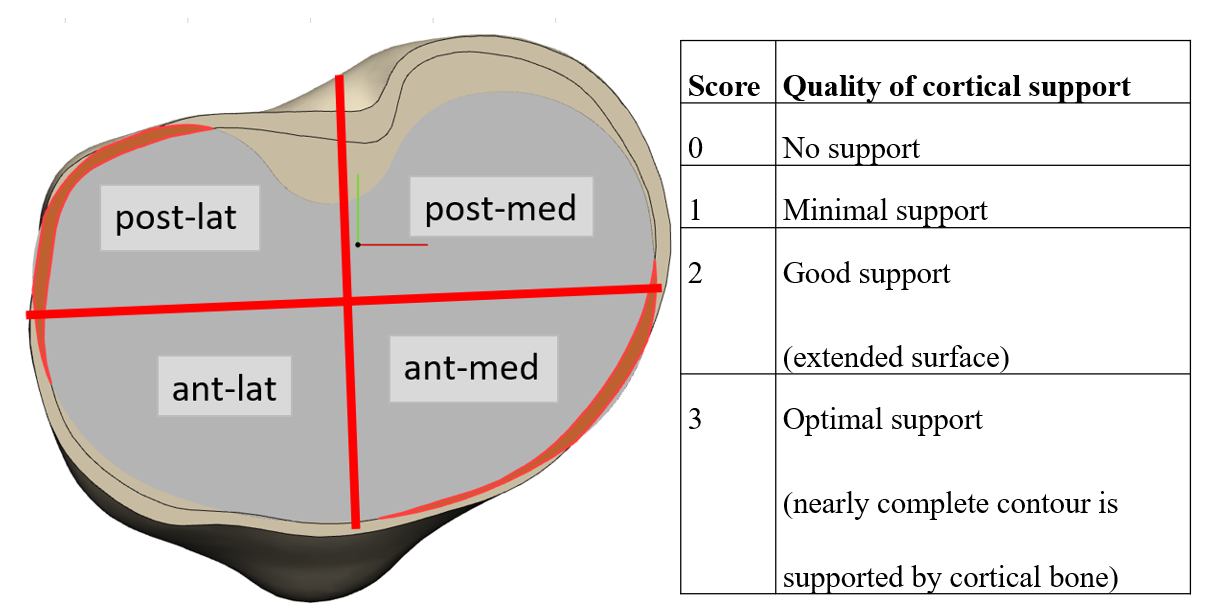

Supplement: S3 Fig — (TIF) [file pone.0354876.s003.tif]

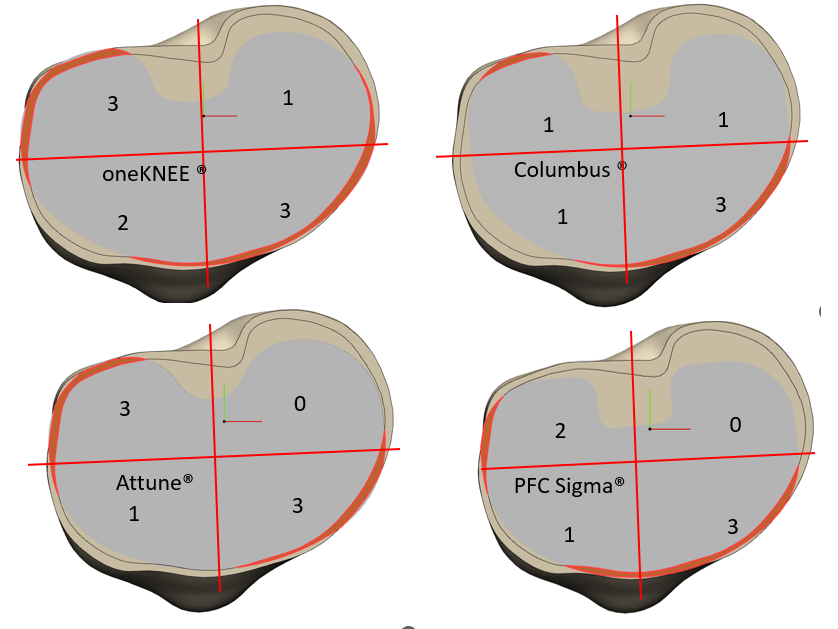

Supplement: S4 Fig — The semi-quantitative score (0–3) is shown for each of the four anatomical quadrants. Representative example shown for illustration; conclusions are drawn from the summarized results across all models. (TIF) [file pone.0354876.s004.tif]
